# Liver function tests in patients with hypertension in primary care: a prospective cohort study

**DOI:** 10.3399/BJGPO.2023.0082

**Published:** 2024-02-07

**Authors:** Thuraiya Al Harthi, Penny Whiting, Jessica Watson

**Affiliations:** 1 Research Department, The Royal Hospital, Ministry of Health, Muscat, Oman; 2 Population Health Sciences, Bristol Medical School, University of Bristol, Bristol, UK; 3 Centre for Academic Primary Care, Bristol Medical School, University of Bristol, Bristol, UK

**Keywords:** predictive value, liver function tests, liver diseases, primary health care, general practitioners

## Abstract

**Background:**

Liver function tests (LFTs) are frequently used to monitor patients with hypertension in UK primary care. Evidence is lacking on whether testing improves outcomes.

**Aim:**

To estimate the diagnostic accuracy of LFTs in patients with hypertension and determine downstream consequences of testing.

**Design & setting:**

Prospective study using the Clinical Practice Research Datalink (CPRD).

**Method:**

In total, 30 000 patients with hypertension who had LFTs in 2015 were randomly selected from CPRD. The diagnostic accuracy measures for eight LFT analytes and an overall LFT panel were calculated against the reference standard of liver disease. Rates of consultations, blood tests, and referrals within 6 months following testing were measured.

**Results:**

The 1-year incidence of liver disease in patients with hypertension was 0.5% (95% confidence interval [CI] = 0.4% to 0.6%). Sensitivity and specificity of an LFT panel were modest: 61.3% (95% CI = 53.1% to 69.0%) and 73.8% (95% CI = 73.1% to 74.3%), respectively. The positive predictive value (PPV) of the eight individual LFT analytes were low ranging from 0.2% to 8.9%. Among patients who did not develop liver disease, mean number of consultations, referrals, and tests were higher in the 6 months following false-positives at 10.5, 0.7 and 29.8, respectively, compared with true-negatives: 8.6, 0.6, and 19.8.

**Conclusion:**

PPVs of LFTs in primary care were low, with high rates of false-positive results and increased rates of subsequent consultations, referrals, and blood testing. Avoiding LFTs for routine monitoring could potentially reduce patients’ anxiety, GP workload, and healthcare costs.

## How this fits in

GPs in UK primary care frequently perform liver function tests (LFTs) for routine evaluation of patients with hypertension despite being not recommended by national guidelines. There are no studies that evaluated the benefits and harms of this practice. Our study found that the yield of diagnosis of liver disease following LFT testing in primary care patients with hypertension is low, with high levels of false-positives, and increased rates of follow-up consultation, further testing, and referrals.

## Introduction

Blood testing for chronic disease monitoring is thought to account for more than 50% of all biochemical blood tests in UK primary care.^1^ GPs usually carry out these tests in order to monitor disease progression and response to treatment.^2^ However, this has cost and workload implications for healthcare systems. It has been estimated that the NHS in England carries out 1.2 billion pathology tests each year,^3^ with GPs estimated to spend 1.5–2 hours per day reviewing test results.^4^ GPs usually follow relevant clinical guidelines for chronic disease monitoring, but most of these guidelines are based on expert opinion with a lack of evidence to support recommendations for optimal testing in long-term conditions.^5^ A recent survey of 550 GPs demonstrated a high level of disagreement for whether liver function tests should be done ‘routinely’, ‘sometimes’, or ‘never’ for patients with hypertension, and a lack of confidence in dealing with abnormal results.^6^

Jones *et al* ^7^ evaluated the variation of blood testing for long-term conditions, including hypertension, and found that liver function tests (LFTs) were the second most frequently requested test,^4^ despite not being recommended by clinical guidelines.^8^ Overuse of LFTs could potentially increase the risk of false-positive results, which may cause patient anxiety and potentially unnecessary follow-up appointments, blood tests, or referrals.^5,6^

LFTs comprise a group of up to eight analytes and are usually used to evaluate abdominal or non-specific general symptoms, monitor chronic disease, and screen for systemic and infectious diseases.^2^ Previous studies on LFT accuracy have focused on the general population rather than patients with chronic disease such as hypertension, and found that fewer than 5% of people with abnormal LFT results had a specific disease of the liver, and many of these were unlikely to need treatment.^9^ Other previous studies of LFT accuracy were hospital-based^10,11^ and not specific to primary care.

The aim of this study was to determine the diagnostic accuracy of LFT, as an overall combined panel and as individual analytes, for detecting liver disease in patients with hypertension in UK primary care and to quantify rates of follow-up consultations, referral, and blood tests after testing.

## Method

### Study population

This was a prospective cohort study of UK primary care patients using anonymised, routinely collected data from Clinical Practice Research Datalink (CPRD). We randomly selected 30 000 adults aged ≥18 years with hypertension who were tested for liver function in 2015. The index date was defined as the date when the first LFT was performed in 2015. Patients with pre-existing liver disease before the index date were excluded. Other comorbidities were not excluded to ensure generalisability to UK primary care.

### Index test

The tests of interest were LFTs. We evaluated eight individual analytes and an overall LFT panel. The eight analytes were bilirubin, alkaline phosphatase (ALP), alanine aminotransferase (ALT), aspartate aminotransferase (AST), gamma-glutamyl transpeptidase (GGT), albumin, globulin, and total protein (TP). Supplementary Table S1 shows the codelist used to identify LFTs; Supplementary Table S2 shows the standardised reference ranges for each individual analyte. In clinical practice, an LFT is usually requested as a panel of different combined analytes^12^ rather than an individual analyte test, therefore a binary variable 'LFT panel' was generated; this was defined as abnormal if any one or more of the LTF analytes performed on the index date was outside of the reference range. Sequential testing within 2015 was not examined.

### Target condition

The target condition was liver disease diagnosed within 1-year following the index date. We defined liver disease based on a published code list developed by the London School of Hygiene and Tropical Medicine^13^ (Supplementary Table S3), which was checked by two of the study investigators (TAH and JW), who are GPs, to ensure all codes were relevant to adult liver disease.

### Consequences following liver function testing

The cascade effect following LFT identified in this study were the rates of consultations, referral, blood tests, and visits for blood tests within 6 months after testing for patients with true-positive, false-positive, false-negative, and true-negative results. The definition of test result groups is defined in Table 4.

### Sample size calculation

Sample size was based on estimating the 1-year incidence of liver disease among those with an abnormal LFT. The BALLETS study reported that around 15% of LFTs in primary care are likely to be abnormal.^9^ Using a conservative estimate of 10% test-positive and assuming an incidence of 3% at baseline in the normal (negative) test group, a total sample size of 20 160 would be required to achieve 90% power at a level of significance of 5% to detect an increase of 2% in condition incidence in those with positive tests compared with the negative test group. A total sample size of 30 000 patients was requested to allow for sub-group analyses based on age, sex, and LFT analytes, and to allow for dropouts after applying the exclusion criteria.

### Statistical analysis

Baseline characteristics were described for the overall cohort and stratified according to LFT panel result. Test results for individual analytes and overall LFT panels were cross-classified against presence or absence of the liver disease to generate 2 × 2 tables of test performance from which sensitivity, speciﬁcity, positive predictive value (PPV), negative predictive value (NPV), and diagnostic odds ratios (DOR) were calculated. Test results were also treated as continuous variables after logarithmic transformation, owing to their skewed distribution, to calculate the area under the receiver operator characteristic (ROC) curve (AUC). We used test consequence graphics to demonstrate the implications of cascade effects following testing on patients’ health outcomes.^14^ Sensitivity analysis was conducted as described in Supplementary Box S1.

We did not use imputation to investigate the impact of missing data, as we cannot be sure that these data are missing at random. Instead, we described how much data were missing. The reporting of this study followed the Standards for Reporting of Diagnostic Accuracy Studies (STARD)^15^ and the REporting of studies Conducted using Observational Routinely collected health Data (RECORD)^16^ guidance. Analyses were performed using Stata (version 16).

## Results

**Figure 1.**
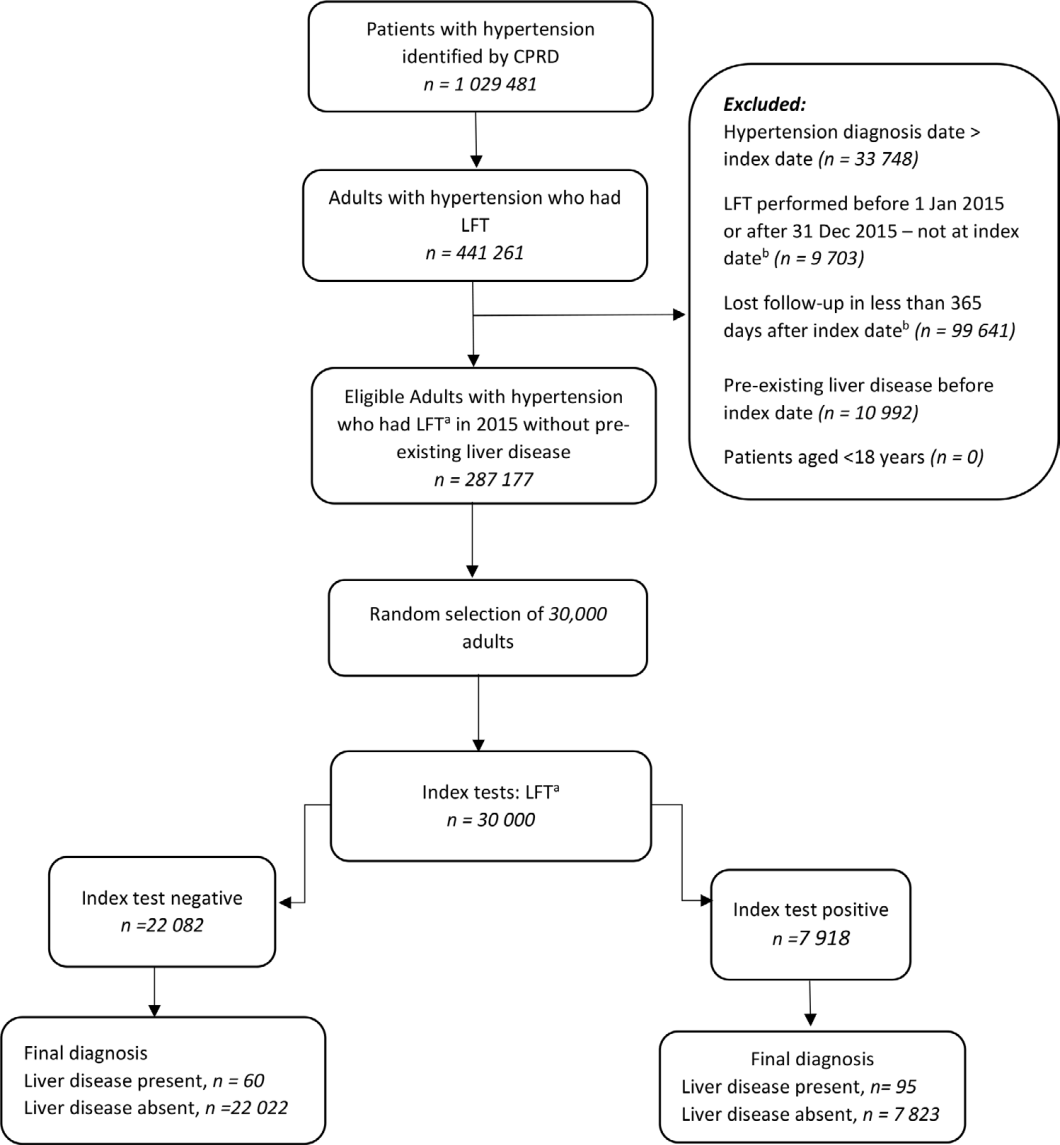
Flow of participants through study. CPRD = Clinical Practice Research Datalink. LFT = liver function test. ^a^LFT panel of two or more analytes that is defined as abnormal if any of the analytes not within the reference range. ^b^The date of the first liver function test done in 2015.

This study included 30 000 patients with hypertension who underwent LFT in 2015. Figure 1 illustrates patient flow from the original source, after exclusion and random selection by the CPRD to form the eligible study cohort. Fifty-two per cent of the study sample were female. The median age at the index date was 69.6 years, with an interquartile range (IQR) of 78.2–60.6 years.

### Description of index test

All patients had more than one analyte requested simultaneously (LFT panel) and there was variation in the type of analytes performed. Supplementary Table S4 shows the frequencies of LFTs from highest to least requested. Only 206 (0.7%) patients had all eight LFT analytes performed together. Table 1 shows baseline characteristics for the overall patient cohort and stratified by LFT panel result. Missing data for test results were rare (Supplementary Box S2).

**Table 1.** Baseline characteristics by liver function test panel of more than one analyte^a^

	Abnormal LFT*^b^*	Normal LFT*^b^*	Total
Number of patients, *n* (%)	7918 (26.4%)	22 082 (73.6%)	30 000 (100%)
Male, *n* (%)	4267 (29.4%)	10 257 (70.6%)	14 524 (100%)
Female, *n* (%)	3651 (23.6%)	11 825 (76.4%)	15 476 (100%)
Age, (median, IQR), years	68.7 (78.1–59.0)	69.8 (78.2–61.3)	69.5 (78.2–60.6)
<40 years, *n* (%)	144/444 (32.4%)	300/444 (67.6%)	444 (100%)
40–50 years, *n* (%)	553/1885 (29.3%)	1332/1885 (70.7%)	1885 (100%)
50–60 years, *n* (%)	1457/4842 (30.1%)	3385/4842 (70.0%)	4842 (100%)
60–70 years, *n* (%)	2121/8296 (25.6%)	6175/8296 (74.4%)	8296 (100%)
70–80 years, *n* (%)	1999/8551 (23.4%)	6552/8551 (76.6%)	8551 (100%)
>80 years, *n* (%)	1644/5982 (27.5%)	4338/5982 (72.5%)	5982 (100%)
Liver disease diagnosed in 1 year (*n*)	95	60	155
Incidence of liver disease within 1 year % (95% confidence interval)	1.20% (0.97% to 1.46%)	0.27% (0.21% to 0.35%)	0.52% (0.44 % to 0.60%)

^a^Baseline characteristics including age, sex, and liver disease incidence sorted by LFT panel of more than one analyte.^b^LFT panel with more than one analyte is defined as abnormal if any of the analytes are not within the reference range. Of all 30 000 patients, 7918 (26.4%) had at least one abnormal test result, which was more frequent in males compared with females (*P*<0.001). The median age was 68.7 years in those with normal LFT and 69.8 years in abnormal test. For categorical age groups, a higher proportion of patients <40 years had abnormal tests compared with older age groups, *P<0.001*. CI = confidence interval.

IQR = interquartile range. LFT = liver function test.

### Incidence of liver disease (target condition)

The 1-year incidence of liver disease was 0.5% (*n* = 155/30 000). Incidence was higher in those with at least one abnormal test; 1.20% (95% CI = 1.0% to 1.5%) compared with 0.3% (95% CI = 0.2% to 0.4%) in those with normal test results (*P*<0.01). The incidence of liver disease across LFTs was highest in patients who had positive AST and GGT tests (Table 2). The type of liver disease most frequently diagnosed was fatty liver disease (Table 3).

**Table 2.** Diagnostic accuracy measures of liver function tests for liver disease diagnosis in patients with hypertension in primary care

Liver function tests (analytes)	Prevalence %(95% CI)	Sens %(95% CI)	Spec %(95% CI)	PPV %(95% CI)	NPV %(95% CI)	DOR(95% CI)
^a^LFT panel of two or more analytes performed simultaneously (*n* = 30 000)	0.5(0.4 to 0.6)	61.3(53.1 to 69.0)	73.8(73.1 to 74.3)	1.2(1.0 to 1.5)	99.7(99.7 to 99.8)	4.5(3.2 to 6.2)
Albumin (*n* = 28 988)	0.5(0.4 to 0.6)	5.5 (2.4 to 10.6)	94.5(94.2 to 94.8)	0.5(0.2 to 1.0)	99.5(99.4 to 99.6)	1.0(0.5 to 2.0)
Bilirubin (*n* = 29 189)	0.5(0.4 to 0.6)	8.0(4.2 to 13.6)	96.2(95.9 to 96.4)	1.1(0.6 to 1.9)	99.5(99.4 to 99.6)	2.2(1.2 to 3.9)
Globulin (*n* = 11 557)	0.4(0.3 to 0.6)	2.1(0.1 to 11.1)	94.6(94.2 to 95.0)	0.2(0.0 to 0.9)	99.6(99.4 to 99.7)	0.4(0.0 to 2.1)
TP (*n* = 18 376)	0.4(0.4 to 0.5)	6.2(2.0 to 13.8)	97.4(97.2 to 97.6)	1.0(0.3 to 2.4)	99.6(99.5 to 99.7)	2.5(1.0 to 5.9)
ALP (*n* = 29 572)	0.5(0.4 to 0.6)	19.0(13.1 to 26.1)	94.8(94.5 to 95.1)	1.9(1.3 to 2.7)	99.6(99.5 to 99.6)	4.3(2.9 to 6.4)
AST (*n* = 4294)	1.0(0.7 to 1.4)	47.2(30.4 to 64.5)	95.1(94.4 to 95.8)	8.9(5.3 to 13.9)	99.4(99.1 to 99.7)	17.4(8.9 to 33.8)
ALT (*n* = 27 738)	0.5(0.4 to 0.6)	29.1(21.7 to 37.3)	95.9(95.6 to 96.1)	3.5(2.5 to 4.7)	99.6(99.5 to 99.7)	9.5(6.6 to 13.8)
GGT (*n* = 8282)	1.0(0.8 to 1.2)	79.3(68.9 to 87.4)	60.5(59.4 to 61.5)	2.0(1.5 to 2.5)	99.7(99.5 to 99.8)	5.9(3.4 to 9.9)

^a^LFT panel of more than one analyte that is defined as abnormal if any of the analytes not within the reference range.

ALP = alkaline phosphatase. ALT = alanine aminotransferase. AST = aspartate aminotransferase. CI = confidence interval. GGT = gamma-glutamyl transpeptidase. LFT = liver function test. NPV = negative predictive value, probability of not having a target condition if test is negative. PPV = positive predictive value, probability of having a target condition if test is positive. Sens = sensitivity. Spec = specificity. TP = total protein.

**Table 3.** Types and frequency of liver diseases diagnosed within 1 year following liver function test

Type of liver disease^a^	Liver disease diagnosis (frequency)	Frequency (%)
Fatty liver disease	Non-alcoholic fatty liver (66), Fatty change of the liver (50), Fatty liver (7), Alcoholic fatty liver (3)	126 (81.3)
Cirrhosis and liver fibrosis	Cirrhosis and chronic liver disease (9), Cirrhosis — non-alcoholic (1), Hepatic fibrosis (1), Portal hypertension (1)	12 (7.7)
Liver disease, unspecified^b^	Liver disorder NOS (6), Acute liver failure (1), Hepatosplenomegaly (1)	8 (5.2)
Alcohol-related liver disease, others^c^	Alcoholic liver damage unspecified (2), Acute alcoholic hepatitis (1), Alcoholic fibrosis and sclerosis of liver (1), Alcoholic hepatitis (1)	5 (3.2)
Viral	Acute hepatitis E (1), Hepatitis B (1)	2 (1.3)
Autoimmune	Autoimmune chronic active hepatitis (1), Primary biliary cirrhosis (1)	2 (1.3)
Total		155 (100)

^a^Liver diseases were categorised based on disease pathology or underlying cause. ^b^No definitive liver diagnosis given. ^c^Liver disease related to alcohol consumption other than alcoholic fatty liver.

NOS = not otherwise specified.

### Diagnostic test accuracy

Table 2 shows measures of diagnostic accuracy for each individual analytes and the overall LFT panel. The positive predictive value (PPV) of the eight individual LFT analytes were low ranging from 0.2% to 8.9%. For most individual analytes, estimates of sensitivity and specificity were <50% and >90%, respectively; the exception was GGT, with sensitivity of 79.3% (95% CI = 68.9% to 87.4%) and specificity of 60.5% (95% CI = 59.4% to 61.5%). For the overall LFT panel, the sensitivity was 61.3% (95% CI = 53.1% to 69.0%) and specificity was 73.8% (95% CI = 73.1% to 74.3%). The DOR suggested increased odds of liver disease if at least one analyte was abnormal compared with a normal LFT panel DOR 4.5 (95% CI = 3.2 to 6.2), *P*<0.001.

Supplementary Figure S1 shows the AUC for LFTs. For the individual analytes the AUC was highest for AST 0.80 (95% CI = 0.72 to 0.89) followed by GGT 0.79 (95% CI = 0.75 to 0.85), ALT 0.78 (95% CI = 0.73 to 0.82), and ALP 0.63 (95% CI = 0.58 to 0.68), indicating modest increase in correctly detecting liver disease. The protein-based LFTs and bilirubin had lower accuracy with AUCs of 0.57 (95% CI = 0.52 to 0.64) for TP, 0.54 (95% CI = 0.46 to 0.62) for globulin, 0.49 (95% CI = 0.45 to 0.55) for albumin, and 0.54 (95% CI = 0.49 to 0.58) for bilirubin (Supplementary Figures S1a and S1b).

### Cascade effects following liver function testing

**Figure 2.**
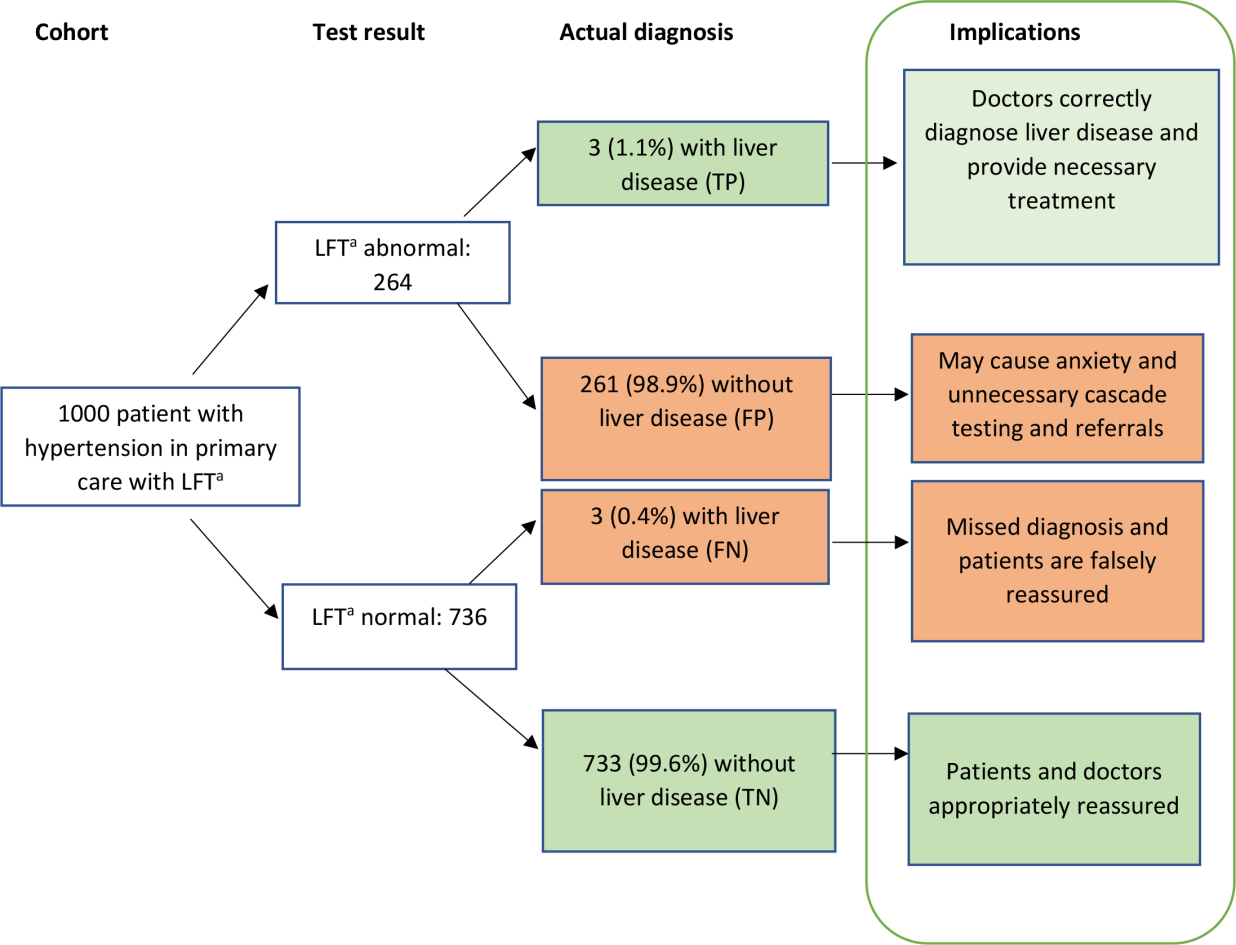
Test implications flowchart. Results that would be obtained if a hypothetical cohort of 1000 patients with hypertension were tested. FN = false-negative. FP = false-positive. LFT = liver function test. TN = true-negative. TP = true-positive.

Table 4 shows the rates of consultations, referrals, blood tests, and GP visits for blood tests within 6 months after LFT testing, for true-positive, false-negative, false-positive, and true-negative patient groups. Among patients who did not develop liver disease, mean number of consultations, referrals, and tests were higher in the 6 months following false-positives at 10.5, 0.7 and 29.8, respectively, compared with true-negatives: 8.6, 0.6, and 19.8. Both groups consisted of patients without liver disease, the main difference being the abnormal LFT result in the false-positive group. Figure 2 shows a graphical illustration of testing implications on a hypothetical cohort of 1000 patients. We would expect 1000 LFT tests to lead to 261 false-positive cases, associated with an additional 496 follow-up consultations, 26 referrals, and 209 follow-on blood test appointments.

**Table 4.** Cascade effects within 6 months after testing using two or more analytes simultaneously^a^

	Mean number of consultations per person^b^ (95% CI)	Mean number of referrals per person (95% CI)	Mean number of total blood tests requested (95% CI)	Mean number of appointments for blood testing in 6 months (95% CI)
True-positive(*n* = 95)	12.4 (10.5 to 14.3)^c^	1.6 (1.2 to 2.0)^c^	51 (36.9 to 65.1)^c^	4.7 (3.7 to 5.7)^c^
False-negative(*n* = 60)	10.3 (8.7 to 11.9)^c^	1.4 (0.8 to 2.0)^c^	33 (22.5 to 43.5)^c^	3.8 (2.9 to 4.7)^c^
False-positive(*n* = 7823)	10.5 (10.2 to 10.7)^c^	0.7 (0.6 to 0.7)^c^	29.8 (28.8 to 30.8)^c^	3.1 (3.0 to 3.2)^c^
True-negative(*n* = 22 022)	8.6 (8.5 to 8.7)^c^	0.6 (0.5 to 0.6)^c^	19.8 (19.3 to 20.2)^c^	2.3 (2.2 to 2.3)^c^

^a^LFT panel of more than one analyte that is defined as abnormal if any of the analytes not within the reference range. True-positives are patients with a positive test who develop target condition. False-positives are patients with a positive test without target condition. False-negatives are patients with a negative test who develop target condition. True-negatives are people with a negative test with no relevant disease. ^b^Includes face-to-face consultations, home visits, and telephone consultations. ^c^ *P*<0.001, comparing true-positives to false-negatives and false-positives to true-negatives

### Sensitivity analysis

Result of sensitivity analyses findings are displayed in Supplementary Tables S5, S6, and S7. Minimal differences in test accuracy were found when using laboratory-specified reference ranges, restricting abnormal test results for albumin, globulin, and TP to test results below the lower limit of normal, and restricting analyses to young adults (aged <40 years), although precision was reduced.

## Discussion

### Summary

This study found 26.4% of patients with hypertension who had a LFT had an abnormal result; however of these, only 1.2% of these were subsequently diagnosed with liver disease. An LFT panel had a modest sensitivity of 61.3% and specificity of 73.8% for liver disease; it is therefore not appropriate either as a rule-out or a rule-in test. The PPV is also low, indicating high rates of false-positive results. Abnormal LFTs (both those with a true-positive and a false-positive result) were associated with increased rates of follow-up consultations, blood tests, and referrals compared with those with a true-negative result. GGT was among the tests that had the highest sensitivity, 79.3% (95% CI = 68.9% to 87.4%). This could be owing to selection bias, as GGT is usually used in patients at higher risk of liver disease, particularly alcoholic liver disease, which might also explain why this test was also the least used test in our cohort.

Among those diagnosed with liver disease, fatty liver was the most common diagnosis, which is a benign condition in its early stage and requires behaviour modification rather than urgent medical intervention.^17^

### Strengths and limitations

The strength of this study relies on the large sample size and the setting in UK primary care, where hypertension is primarily treated and managed. Patients with comorbid diseases were not excluded to ensure generalisability of the results. A test consequence graphic was used to demonstrate the implications of LFT on a hypothetical cohort in order to make results clearer and more clinically relevant. The main limitation is that the reference standard, CPRD-coded diagnosis of liver disease is reliant on the quality of primary care diagnosis and coding of liver disease. Although diagnoses are generally well recorded in CPRD,^18^ liver disease is known to be underdiagnosed, especially in early stages.^19^ Given that non-alcoholic fatty liver disease is estimated to affect 25–35% of the global population^20^ and alcohol-related liver disease affects around 5%,^14^ it is highly unlikely that only 0.5% of this sample had chronic liver disease. The benefit of this approach is that it demonstrates what proportion of LFT-tested patients with hypertension actually receive a diagnosis of liver disease in a primary care setting.

Another limitation is lack of information about the reasons why GPs ordered LFT in patients with hypertension. It is not known whether the test was done for routine monitoring or to test specifically for liver disease owing to the patient presenting with symptoms. There may indeed be good clinical reasons for some LFT tests; however, this doesn't change our finding that the yield of testing is low. Finally, we used standardised reference ranges to define an abnormal test result in this study, whereas in clinical practice thresholds for abnormality differ by hospital, country, and community; sensitivity analysis was conducted showing minimal differences in accuracy using within study laboratory thresholds.

### Comparison with existing literature

Previous studies based on the general population, rather than those with chronic disease, found that 21.7% of those tested had at least one abnormal LFT and 1.2% developed liver disease.^21^ One study in a US internal medicine clinic identified 39% with abnormal LFTs; the higher prevalence presumably reflecting higher rates of comorbidities in a secondary care setting.^22^ In our cohort of patients with hypertension in primary care, 26.4% had at least one abnormal test and the incidence of liver disease within 1 year was 0.5%. The higher prevalence of abnormal results seen in our study compared with the general population might be explained by the multimorbid nature of hypertension that could affect liver enzymes.^12^ The slighter lower incidence of liver disease in our cohorts probably reflects the low frequency of serious disease encountered in primary care settings, but could also reflect underdiagnosis of liver disease in primary care. Another prospective cohort study evaluated abnormal LFT in 1290 primary care patients; all patients with abnormal LFTs were extensively investigated for underlying causes; even with thorough investigation, fewer than 5% of people with abnormal LFT results had a specific disease of the liver, and many of these were unlikely to need treatment.^9^ None of these previous studies explored the downstream consequences of LFT in patients with hypertension.

### Implications for practice

LFTs are not suitable for ruling-in or ruling-out liver disease in patients with hypertension and are not currently recommended for routine monitoring of hypertension in National Institute for Health and Care Excellence (NICE) guidelines.^5^ We found that a relatively high proportion of LFT panels had abnormalities, many subsequent tests, consultations, and referrals were generated, yet few diagnoses were made. Reducing LFT testing could potentially reduce unnecessary downstream referrals, testing, and consultations, with significant cost and workload implications for the NHS.

GPs should consider the limited diagnostic accuracy and potential cascade effects of LFTs and use these tests when there is a clear indication.

Further studies are needed to explore the symptoms and conditions that prompt GPs to request an LFT test for patients with hypertension, other chronic conditions and multimorbidity, and to evaluate the diagnostic accuracy of LFT for these patient groups in primary care.

## Data

This study is based on data from the Clinical Practice Research Datalink (CPRD) obtained under licence from the MHRA and NIHR. The data are provided by patients and collected by the NHS as part of their care and support. CPRD data can be accessed via the MHRA: https://www.cprd.com/
